# Absence of PD-L1 expression on tumor cells in the context of an activated immune infiltrate may indicate impaired IFNγ signaling in non-small cell lung cancer

**DOI:** 10.1371/journal.pone.0216864

**Published:** 2019-05-24

**Authors:** Willemijn S. M. E. Theelen, Thomas Kuilman, Katja Schulze, Wei Zou, Oscar Krijgsman, Dennis D. G. C. Peters, Sten Cornelissen, Kim Monkhorst, Pranamee Sarma, Teiko Sumiyoshi, Lukas C. Amler, Stefan M. Willems, Johannes L. G. Blaauwgeers, Carel J. M. van Noesel, Daniel S. Peeper, Michel M. van den Heuvel, Marcin Kowanetz

**Affiliations:** 1 Department of Thoracic Oncology, The Netherlands Cancer Institute, Amsterdam, The Netherlands; 2 Division of Molecular Oncology & Immunology, The Netherlands Cancer Institute, Amsterdam, The Netherlands; 3 Oncology Biomarker Development, Genentech Inc., South San Francisco, United States of America; 4 Biostatistics, Genentech Inc., South San Francisco, United States of America; 5 Core Facility Molecular Pathology & Biobanking, Department of Molecular Pathology, The Netherlands Cancer Institute, Amsterdam, The Netherlands; 6 Division of Pathology, The Netherlands Cancer Institute, Amsterdam, the Netherlands; 7 Department of Pathology, University Medical Centre Utrecht, Utrecht, The Netherlands; 8 Department of Pathology, OLVG, Amsterdam, The Netherlands; 9 Department of Pathology, Academic Medical Center, Amsterdam, The Netherlands; 10 Department of Pulmonology, Radboud University Medical Center, Nijmegen, The Netherlands; University of Colorado Denver, UNITED STATES

## Abstract

**Background:**

In non-small cell lung cancer (NSCLC), PD-L1 expression on either tumor cells (TC) or both TC and tumor-infiltrating immune cells (IC) is currently the most used biomarker in cancer immunotherapy. However, the mechanisms involved in PD-L1 regulation are not fully understood. To provide better insight in these mechanisms, a multiangular analysis approach was used to combine protein and mRNA expression with several clinicopathological characteristics.

**Patients and methods:**

Archival tissues from 640 early stage, resected NSCLC patients were analyzed with immunohistochemistry for expression of PD-L1 and CD8 infiltration. In addition, mutational status and expression of a selection of immune genes involved in the PD-L1/PD-1 axis and T-cell response was determined.

**Results:**

Tumors with high PD-L1 expression on TC or on IC represent two subsets of NSCLC with minimal overlap. We observed that PD-L1 expression on IC irrespective of expression on TC is a good marker for inflammation within tumors. In the tumors with the highest IC expression and absent TC expression an association with reduced IFNγ downstream signaling in tumor cells was observed.

**Conclusions:**

These results show that PD-L1 expression on TC and IC are both independent hallmarks of the inflamed phenotype in NSCLC, and TC-negative/IC-high tumors can also be categorized as inflamed. The lack of correlation between PD-L1 TC and IC expression in this subgroup may be caused by impaired IFNγ signaling in tumor cells. These findings may bring a better understanding of the tumor-immune system interaction and the clinical relevance of PD-L1 expression on IC irrespective of PD-L1 expression on TC.

## Introduction

One of the most studied tumor immune escape mechanisms is mediated through the inhibitory programmed death-ligand 1 (PD-L1)/programmed death 1 (PD-1) pathway. The development of anti-PD-L1/PD-1 monoclonal antibodies has led to long-lasting anti-tumor immune responses in a subset of patients with non-small cell lung cancer (NSCLC). High PD-L1 expression as assessed by immunohistochemistry (IHC) has consistently been reported to be associated with higher responses to anti-PD-L1/PD-1 treatment, resulting in the development of various diagnostic PD-L1 IHC assays [1–3]. The use of various diagnostic PD-L1 IHC assays has led to ambiguity as to how to use this multi-faceted biomarker. In two randomized trials comparing the anti-PD-L1 antibody atezolizumab to docetaxel in second line setting, PD-L1 expression on TC and on infiltrating immune cells (IC) both appeared to be independently associated with response to atezolizumab [3, 4].

Besides PD-L1 expression, wider aspects of the tumor/immune-infiltrating complex are under investigation as biomarkers for immunotherapy. Tumors can broadly be divided into inflamed (hot) vs non-inflamed (cold) tumors. Typically, inflamed tumors show a pre-existing antitumor immune response with abundance of tumor-infiltrating lymphocytes (TILs), IFNγ-producing CD8^+^ T-cells and high expression of PD-L1. In contrast, non-inflamed tumors are characterized as immune desert: containing hardly any TILs and rarely expressing PD-L1 [5, 6]. The development of gene expression profiling of tumors allows distinguishing ‘hot’ and ‘cold’ tumors by providing prognostic and predictive immune signatures; one example being the T-effector (T_eff_) signature showing an association with efficacy in the randomized phase II and III trials comparing atezolizumab to docetaxel [3, 4].

Hence, it is important to improve insights in the overlap and differences between PD-L1 expression on TC and/or IC and to relate this expression to other tumor features and markers of the PD-L1/PD-1 axis and T-cell response. In order to do this, we used a multiangular approach by combining protein and mRNA expression with clinicopathological characteristics, including mutational analysis of well-known drivers of NSCLC in a large cohort of clinically annotated resected NSCLC samples.

## Material and methods

### Sample collection and patient cohort

Inclusion criteria for this cohort were patients that had undergone a lung resection between 1990 and 2013 at one of four Dutch medical centers. Exclusion criteria were a synchronous primary tumor, unavailability of tumor tissue or patient follow-up data, histology of non-NSCLC, e.g. SCLC or metastasized non-NSCLC. Clinical data about gender, smoking status, neo-adjuvant and adjuvant treatment, age at resection, type of resection, tumor stage, progression free survival (PFS) and overall survival (OS) were collected. No data on treatment after relapse of disease was available. The cohort included 768 samples with adequate patient and tumor characteristics. For all these patients, formalin-fixed, paraffin-embedded (FFPE) tumor samples were collected. After a second pathology revision, samples without sufficient vital tumor material were excluded, leaving 640 samples eligible for further analysis. All tumors were histopathologically classified according the 2015 WHO classification system [7]. TNM classification was redefined for resections that were done before 2010 according to the 7th lung cancer TNM classification and staging system. Smoking status was defined by pack years (PY). Light smokers were defined by having less than 10 PY, including never smokers. Prior to analysis the samples were de-identified. The Translational Research Board of the Netherlands Cancer Institute-Antoni van Leeuwenhoek hospital approved the use of patient data and material in this study.

### Immunohistochemical staining, mutational and gene expression analysis

**Fig 1:**
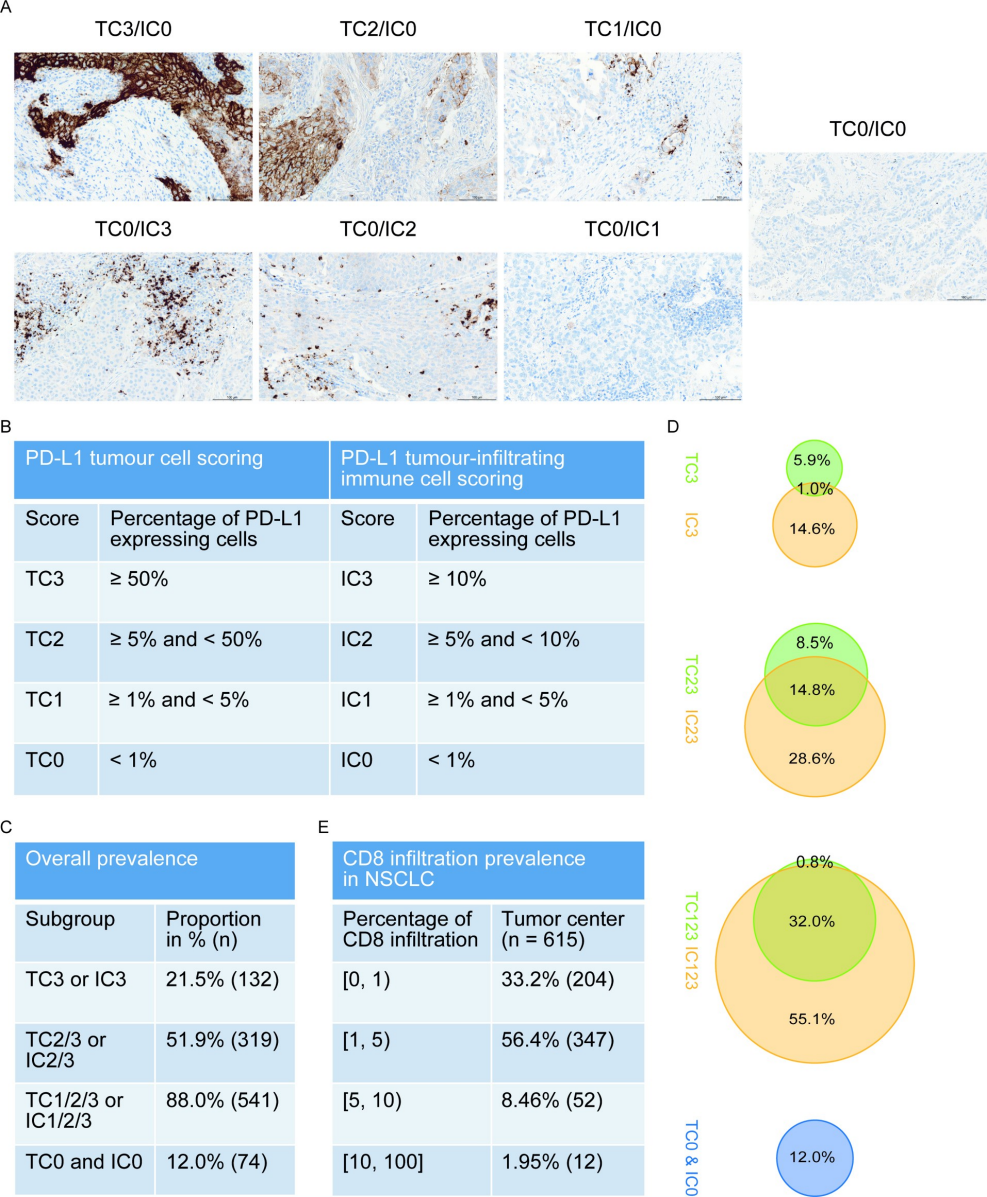
Examples of PD-L1 staining, scoring criteria, prevalence and overlap between PD-L1 expression on TC and IC and prevalence of CD8 infiltration in the tumor center in NSCLC. (A) PD-L1 expression by IHC on both TC and IC for each subgroup. (B) PD-L1 IHC scoring criteria on TC and IC (3) (C) Overall prevalence of overlapping PD-L1 subgroups. (D) Percentages in Venn diagrams represent the prevalence of PD-L1 expression by TC and IC in overlapping subgroups. (E) Overall prevalence of CD8 infiltration in the tumor center.

PD-L1 expression and CD8 staining was assessed in a central laboratory (HistoGeneX, Belgium) using whole slide sections prepared from FFPE resection specimens. Sections were stained using the rabbit anti-human anti-PD-L1 antibody (clone SP142, Spring Bioscience) and the monoclonal mouse anti-human anti-CD8 antibody (clone C8/144B, DAKO) on a Ventana BenchMark XT autostainer (Ventana Medical Systems). PD-L1 expression in TC was assessed as the proportion of TC showing membrane staining of any intensity; expression in IC was assessed as the proportion of tumor area occupied by PD-L1-positive IC of any intensity (Figs 1A and 1B and S1) [3, 4]. In all specimens, total immune infiltrate and tumor cells were assessed in the tumor area by a certified pathologist based on hematoxylin background staining of the IHC slide and if needed based on the H&E staining. Positive and negative controls were performed using tonsil tissue. The scoring algorithm was developed for the approved VENTANA PD-L1 (SP142) Assay and further details concerning the PD-L1 staining protocol have been described previously [8, 9]. PD-L1 score for expression on TC and IC was available for 615 (96.1%) samples. CD8 staining was reported as the percent CD8-positive tumor infiltrating immune cells in the tumor center, available for 615 (96.1%) samples.

Mutation analysis was performed using a microfluidics-based PCR platform running an allele-specific multiplex test as previously described [10, 11]. The validated panel included a total of 130 hot spot mutations (Table A in S1 File). Immunohistochemistry for *ALK* was performed on a BenchMark Ultra autostainer (Ventana Medical Systems) using clone 5A4 (Abcam). For *ALK* FISH staining and analysis of the results was performed as described by the manufacturer.

DNA was extracted using the Qiagen DNA mini kit (cat. No. 51306) and a minimum of 80ng DNA was shipped to Genentech Inc. for mutation analysis. Gene expression analysis was performed using the NanoString nCounter Analysis system (NanoString) on 80-200ng RNA extracted from FFPE tissue samples. A customized gene panel, including 795 targets including multiple genes of immunologic function and cancer biology and including 4 housekeeping genes was applied. Following thorough assay quality control, data were normalized and underwent analysis. We report here results for CD8 (*CD8A)*, PD-L1 *(CD274)*, PD-1 (*PDCD1*), PD-L2 (*PDCD1LG2*) and T_eff_ signature that was defined as the mean expression for *CD8A*, *GZMA*, *GZMB*, *IFNG*, *EOMES*, *CXCL9*, *CXCL10* and *TBX21* as previously described [3, 4]. The downstream IFNγ response signature was derived from the DER_IFN_GAMMA_RESPONSE_UP gene set (MSigDB; http://software.broadinstitute.org/gsea/msigdb; 71 genes), where signature expression was calculated by summing the log2-based expression values for genes that are members of the gene set and that are present in the expression data (22 / 71 genes). To calculate the actual minus the expected (residual) IFNγ signature expression, a linear model based on all samples was created describing the relationship between downstream IFNγ response signature and T_eff_ signature expression. This model was used to calculate the expected IFNγ signature expression. Biomarker high and low subgroups were defined by expression levels at or above various cut-offs, either above or below the median or above or below the 25% or 75% quantile. Gene expression analysis was available for 530 (82.8%) of the samples.

### Statistical analysis

All statistical tests were performed in R. Kaplan–Meier methodology was used to construct survival curves. Stratified Cox regression models were used to estimate HRs and 95% CIs in biomarker subgroup populations. For comparison of gene expression data among subgroups, (pairwise) t-tests were performed. For comparison of protein expression data among subgroups, (pairwise) Wilcoxon rank sum tests were performed. For comparison of categorical data among subgroups, Fisher's exact tests or Pearson's Chi-squared tests were used as indicated. The false discovery rate (FDR) was controlled below 0.05 using Benjamini-Hochberg method.

## Results

### Description of the cohort and distribution of PD-L1 protein expression and CD8 infiltration

The cohort consisted of 640 NSCLC samples: 344 (53.8%) AC, 267 (41.8%) SCC and 29 (4.5%) NSCLC NOS. Only 48 (7.5%) patients were light or never smokers. 83.9% of the cohort was early stage disease (≤ stage II). Median follow-up time was 96.0 months (95% CI: 86–103). All samples were screened for presence of an *ALK* translocation or mutations of well-known drivers in NSCLC. Mutational analysis was available for 563 (88.0%) samples: 170 mutations were found in 164 patients (29.1%). Six samples harbored two mutations. *ALK* IHC was available for 630 (98.4%) samples. Four samples (0.6%) were *ALK* IHC positive and a translocation was confirmed by FISH. *EGFR*, *KRAS*, *BRAF* and *ALK* aberrations were mutually exclusive. Table 1 summarizes the clinicopathological characteristics and genetic alterations of our patient cohort.

**Table 1:** Patients’ and tumor characteristics of the non-small cell lung cancer cohort.

	AC	SCC	NSCLC NOS
Total (n = 640)	344	267	29
**Gender**			
Male	163 (47.3%)	188 (70.4%)	17 (58.6%)
Female	181 (52.7%)	79 (29.6%)	12 (41.4%)
**Median age at surgery (years, range)**	62 (30–84)	67 (38–85)	57 (37–81)
**Neo-adjuvant therapy**	54 (15.7%)	13 (4.9%)	7 (24.1%)
Chemotherapy	21 (6.1%)	2 (0.7%)	1 (3.4%)
Concurrent chemo radiotherapy	8 (2.3%)	2 (0.7%)	4 (13.8%)
Sequential chemo radiotherapy	3 (0.9%)	0	0
Erlotinib [12]	22 (6.4%)	6 (2.2%)	2 (6.9%)
Radiotherapy	0	3 (1.1%)	0
No neo-adjuvant therapy	290 (84.3%)	254 (95.1%)	22 (75.9%)
**Adjuvant treatment**			
Chemotherapy	49 (14.2%)	45 (16.9%)	8 (27.6%)
Radiotherapy	19 (5.5%)	24 (9.0%)	2 (6.9%)
Chemotherapy + radiotherapy	7 (2.0%)	9 (3.4%)	1 (3.4%)
No adjuvant therapy	244 (70.9%)	160 (59.9%)	14 (48.3%)
Unknown	25 (7.3%)	29 (10.9%)	4 (13.8%)
**Smoking**			
Light smokers <10PY	42 (12.2%)	4 (1.5%)	2 (6.9%)
Heavy smokers ≥10PY	253 (73.5%)	224 (83.9%)	25 (86.2%)
Unknown	49 (14.2%)	39 (14.6%)	2 (6.9%)
**Tumor stage at resection**			
Stage I	211 (61.3%)	131 (49.0%)	13 (44.8%)
Stage II	79 (23.0%)	95 (35.6%)	9 (31.0%)
Stage III	44 (12.8%)	34 (12.7%)	7 (24.1%)
Stage IV	10 (2.9%)	7 (2.6%)	0
Genetic alterations*			
EGFR mutated	20 (6.3%)	1 (0.5%)	0
KRAS mutated	110 (34.6%)	7 (3.4%)	3 (10.3%)
ALK translocated	4 (1.3%)	0	0
PIK3CA mutated	10 (3.1%)	14 (6.8%)	0
BRAF mutated	1 (0.3%)	0	0
NRAS mutated	1 (0.3%)	2 (1.0%)	0
HRAS mutated	1 (0.3%)	0	0
No mutation detected	171 (53.8%)	182 (88.3%)	26 (89.7%)
Undetermined^	26	61	0
**Mean overall survival (months, range)**	71 (0–285)	76 (0–289)	71 (6–273)

* percentages for analyzed samples only.

*EGFR* mutations included exon 19 deletions (n = 15), exon 20 insertions (n = 2) and exon 21 L858R mutations (n = 4). No T790M mutations were found. *KRAS* mutations included mutations in codon 12 and 13 (n = 116) and codon 61 (n = 4). Mutations in *AKT1*, *ERBB2*, *FLT3*, *JAK2*, *KIT*, *MYD88* were not present within this cohort. All present *MET* mutations (n = 30) were germline single nucleotide polymorphism (SNP).

^ mutation status was undetermined when no sufficient DNA was available or when the microfluidics-based PCR platform lead to an invalid result.

SCC = squamous cell carcinoma, AC = adenocarcinoma, NSCLC NOS = non-small cell lung cancer not otherwise specified, PY = pack years

In order to investigate the overlap and differences of PD-L1 protein expression between TC and IC, all samples were scored for PD-L1 expression on TC and on IC at all four expression levels. Examples of PD-L1 staining, PD-L1 IHC scoring criteria, the overall prevalence and distribution by overlapping PD-L1 subgroups are presented in Figs 1A–1D and S1. Non-overlapping PD-L1 subgroups are presented in S2 Fig. High PD-L1 expression (TC3 or IC3) was present in 132 (21.5%) samples and 74 (12.0%) samples showed no PD-L1 expression (TC0 and IC0 subgroup) (Fig 1C). Only a minority of samples (10.4%) had CD8 infiltration in the tumor center of 5% or higher (Fig 1E).

Inflammatory features like PD-L1 expression may be affected by traditional stratifying criteria (i.e. gender, age, smoking status, histology, tumor stage or *KRAS*/*EGFR* status). In a univariate analysis using the TC and IC scores separately a positive association between heavy smoking and PD-L1 expression on TC (p = 0.016) was found, but not for PD-L1 expression on IC. There was no significant difference in PD-L1 expression between the histologic subtypes (S3A–S3D Fig). PD-L1 expression on TC was significantly higher for *KRAS* mutant (*KRASm*) tumors compared to *KRAS* wild type (*KRASwt*) tumors (p < 0.001) and this was irrespective of smoking status. No difference was found for PD-L1 expression on IC by *KRAS* status (S3E and S3F Fig). *EGFRm* status was not significantly associated with PD-L1 protein expression (data not shown).

The correlation between PD-L1 protein expression and PD-L1 mRNA expression (encoded by *CD274*) was investigated. Protein expression of both TC and IC was significantly associated with mRNA expression of *CD274* (S4 Fig).

### Overlap and differences of PD-L1 protein expression on TC and IC

**Fig 2:**
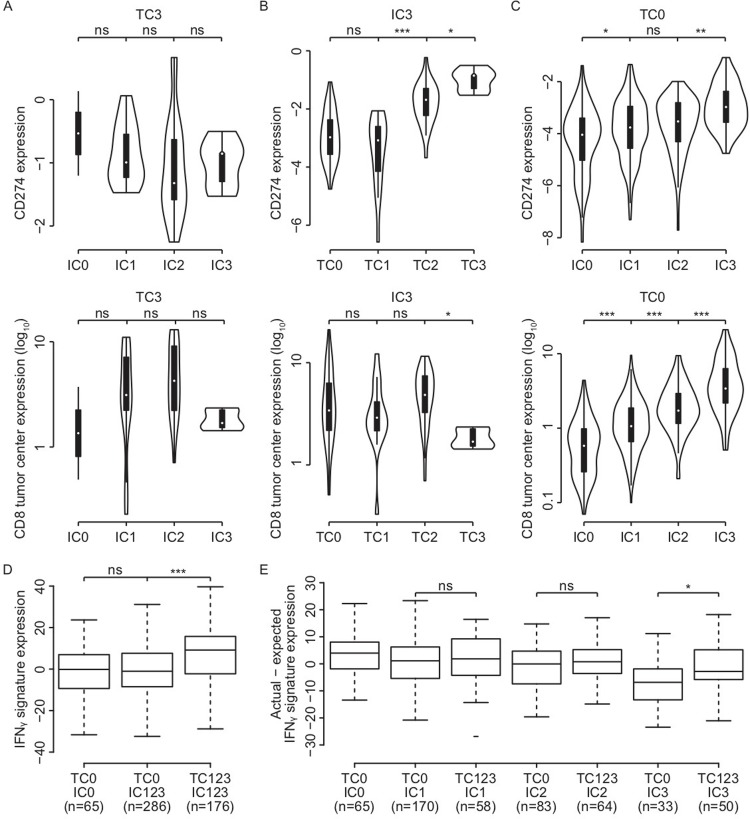
Associations of mRNA expression of *CD274*, infiltration of CD8 and the IFNγ response signature in non-overlapping PD-L1 expressing subgroups. (A) Relative mRNA expression of *CD274* and CD8 infiltration in TC3 tumors based on various levels of IC (n = 39). (B) Relative mRNA expression of *CD274* and CD8 infiltration in IC3 tumors based on various levels of TC (n = 83). (C) Relative mRNA expression of *CD274* and CD8 infiltration in TC0 tumors based on various levels of IC (n = 351). (D) Relative mRNA expression of the IFNγ response signature in non-overlapping PD-L1 subgroups: TC0&IC0, TC0/IC123 and TC123/IC123 (n = 530). (E) The actual minus the expected relative mRNA expression of the IFNγ response signature comparing TC negative to TC positive samples for each non-overlapping IC-subgroup. Expected IFNγ response signature expression was obtained from the level of T_eff_ signature expression based on their linear relationship. ns = non significant, * p = 0.01–0.05, ** p < 0.01, *** p < 0.001.

We then explored the distribution of PD-L1 expression and the overlap and differences between expression on TC and IC. There was minimal overlap between TC3 and IC3 tumors (1.0%, Fig 1D), which might suggest different mechanism of PD-L1 upregulation in tumor cells compared to immune cells. Comparing TC3 tumors to IC3 tumors in regard to clinicopathological features did not reveal significant differences (Table B in S1 File). Next, we analyzed potential differences with respect to immunological features. To correct for potential confounding of the true biology of TC3 and IC3 tumors by the PD-L1 expression in the other compartment, we compared TC3 tumors based on various expression levels of IC (0 to 3) to IC3 tumors based on various expression levels of TC (0 to 3) (Figs 2A–2C and S5). In the TC3 subgroup (n = 39), expression of all inflammatory markers showed a slight increase per increasing IC subgroup except expression of *CD274*, but this was not significant. In the IC3 subgroup (n = 83), we found that only expression of *CD274* increased per increasing TC score, while the other inflammatory markers remained constant, i.e. CD8 infiltration, T_eff_ signature, *CD8A*, *PDCD1* and *PDCD1LG2* expression. Also, when evaluating TC0 samples based on various levels of IC (0 to 3; n = 351) all inflammatory markers, including *CD274*, increased per ascending IC subgroup. The IC score therefore seems to represent a characteristic of true ‘hot’ tumors.

As the TC0/IC123 subgroup (n = 286, 55.1%) contains the majority of samples in this cohort (Fig 1D), we then sought to understand why tumors harboring an active immune infiltrate showed no upregulation of PD-L1 on TC. Since IFNγ signaling is an important mechanism for PD-L1 upregulation, we hypothesized that an impairment in downstream IFNγ signaling within tumor cells might explain this phenomenon. It is expected that cytokine production by an active immune infiltrate, represented by the T_eff_ signature, will lead to downstream IFNγ signaling within tumor cells. Therefore, we determined the expression of selected IFNγ target genes, and collectively represented them as an IFNγ response signature. The expression of this IFNγ response signature was significantly lower in TC negative samples compared to TC positive samples (p < 0.001, Fig 2D). As this difference was irrespective of the expression of the IFNγ target PD-L1 on IC, this strongly suggests that expression of this IFNγ response signature originated from tumor cells only and not the immune infiltrate. The T_eff_ and the IFNγ response signature showed a linear relationship (S6 Fig). We calculated the difference between the expected level of the IFNγ response signature based on this linear model and the actual one (residuals). To overcome confounding by the IC score, again we compared the residuals in TC0 tumors based on various subgroups of IC (0 to 3) (Fig 2E). In the TC0/IC3 subgroup, we observed a significantly lower expression of IFNγ response as would be expected by the linear model compared to the TC123/IC3 subgroup (p = 0.042). Expected expression in the TC0/IC3 subgroup was lower compared to all other subgroups. This suggests that the absence of PD-L1 expression on tumor cells in TC0/IC3 samples may be caused by impaired IFNγ signaling in these tumor cells.

### Prognostic value of PD-L1 expression, CD8 infiltration and gene expression

**Fig 3:**
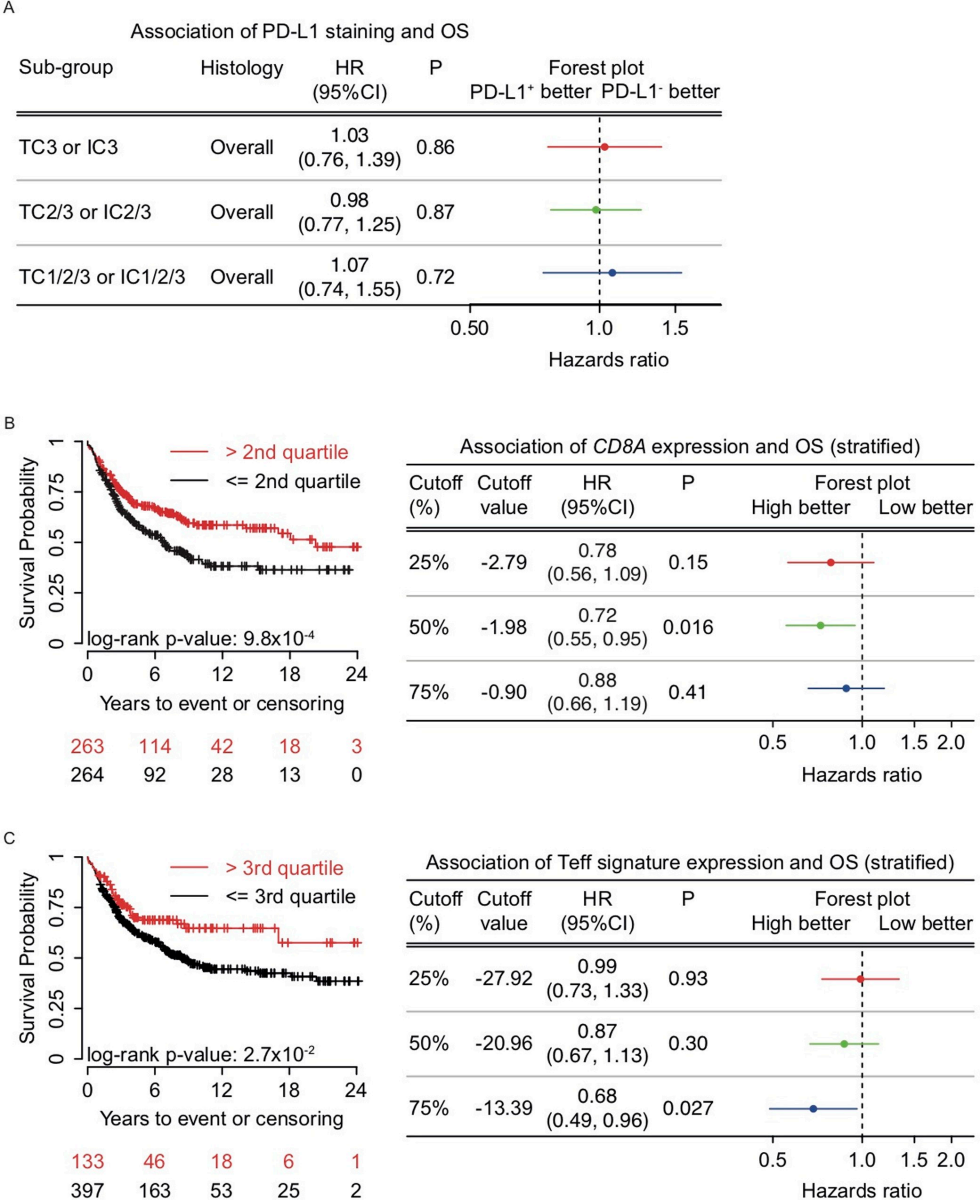
The effect of PD-L1 expression, the expression of *CD8A* and the T_eff_ signature on OS. (A) Forest plot for overlapping PD-L1 expressing subgroups show no improved OS for higher PD-L1 expression; stratified for tumor stage. (B) Forest plot and Kaplan Meier curve for *CD8A* expression show improved OS for the highest two quartiles; stratified for tumor stage (HR 0.72 (95%CI 0.55–0.95; p = 0.016)). (C) Forest plot and Kaplan Meier curve for quartiles of the T_eff_ signature show improved OS for the highest quartile; stratified for tumor stage (HR 0.68 (95%CI 0.49–0.96; p = 0.027)).

Recent data showed conflicting results concerning the prognostic value of PD-L1 expression in NSCLC. After stratifying for tumor stage, we analyzed the prognostic value of several inflammatory parameters measured in our cohort. PD-L1 protein expression -combined or on TC/IC separately- and mRNA expression of *CD274* had no effect on OS in our cohort (Fig 3A). CD8 infiltration by IHC only showed a trend towards improved OS, but *CD8A* transcript levels were significantly associated with better OS (HR 0.72 (95%CI 0.55–0.95; p = 0.016, Fig 3B). High expression of the T_eff_ signature (highest quartile) and *PDCD1* (highest quartile) were both positive prognostic markers (HR 0.68 (95%CI 0.49–0.96; p = 0.027), Fig 3C and HR 0.60 (95%CI 0.42–0.85; p = 0.0035), data not shown, respectively). Expression of *PDCD1LG2* or the IFNγ response signature had no OS relevance. Based on these results, we conclude that gene expression profiling is a better indicator of prognosis than PD-L1 protein expression.

## Discussion

To date, PD-L1 protein expression on tumor cells and on tumor infiltrating immune cells is the most studied biomarker in cancer immunotherapy. This study sought to improve insights in the relation of PD-L1 protein expression with traditional stratifying criteria, like histology and oncogenic driver status, and other markers of the PD-L1/PD-1 axis and T-cell response.

In our cohort, the pattern of inflamed tumors was clearly established: expression of PD-L1 on either TC or IC, infiltration of CD8+ cells and mRNA expression of *CD274*, *CD8A*, *PDCD1*, *PDCD1LG2* and the T_eff_ signature were all associated with one another. Besides this overlap in inflamed features, also differences between PD-L1 expression on TC and IC were found. Co-expression of PD-L1 at the highest level on both TC and IC rarely occurred: prevalence of TC3&IC3 population was only 1%. Fehrenbacher et al. also described this lack of overlap in advanced NSCLC and hypothesized an intrinsic mechanism of PD-L1 upregulation on TC versus an adaptive mechanism on IC [3]. Unfortunately, as opposed to the studies in advanced NSCLC, this early stage cohort contained very few TC123/IC0 samples (< 1%). Therefore, our analyses had several limitations because of the risk of confounding by the PD-L1 expression in the other compartment as we could not compare the true PD-L1 TC positive (TC123/IC0) to the true PD-L1 IC positive (TC0/IC123) tumors. By comparing IC3 tumors based on various levels of TC and TC3 as well as TC0 tumors based on various levels of IC, we observed that inflammatory markers like *CD8A* and the T_eff_ signature correlated most clearly with the IC score. Not unsurprisingly, this was strongest within the TC0 subgroup and shows that the IC score is a good measure for true ‘hot’ tumors.

By dividing our cohort into three non-overlapping subgroups -TC0&IC0, TC0/IC123 and TC123/IC123- we were able to explore other differences between PD-L1 expression on TC vs IC. We found a significantly lower IFNγ response signature expression in TC negative versus TC positive tumors, suggesting an inability of the tumor cells to upregulate PD-L1 in the presence of an active and IFNγ producing immune infiltrate as is represented by expression of the T_eff_ signature. And as expected after performing further analysis between TC negative and TC positive samples in increasing IC subgroups, strong evidence of a hampered IFNγ-PD-L1 axis in tumor cells within the TC0/IC3 subgroup was found. As to our knowledge, this finding has not been described or looked into before. We observed impaired expression of the majority of the individual IFNγ response signature genes (data not shown), implying that the impaired IFNγ signaling in TC0/IC3 tumors is due to alterations at an early level of the pathway: IFNGR or JAK/STAT. Kowanetz et al. found that the TC0/IC3 subgroup had a response rate to the PD-L1 inhibitor atezolizumab of 22%, which was higher than TC0&IC0 tumors (ORR 8%), but lower compared to the TC3/IC0 subgroup (ORR 40%) [4, 13]. Therefore, it would be interesting to investigate if restoring this impairment might improve the benefit on PD-1 blockade in these patients.

For the PD-L1 staining, the SP142 antibody clone was used according to a validated protocol assessing PD-L1 expression on IC in addition to TC [3, 4]. This enabled a thorough assessment of the differences and overlap between PD-L1 expression on TC versus IC. However, in the Blueprint analysis comparing four PD-L1 IHC assays, the SP142 staining differed significantly by producing a weaker staining on TC and fewer PD-L1 positive TCs compared to the other three assays (22C3, 28–8 and SP263), which were similar in the analytical performance [14]. Based on these differences between the assays it’s possible that some of the TC positive tumors may have been unjustly qualified as a TC0 tumor in our cohort in comparison with other PD-L1 assays. This may have resulted in an underestimation of the finding of a hampered IFNγ-PD-L1 axis in our TC0 subgroup and might help explain why we did not find this impairment in the TC0/IC2 or TC0/IC1 subgroup.

In conclusion, these results show the important contribution of PD-L1 expression on IC to identify inflamed tumors. Impaired IFNγ response signaling in tumor cells may explain the absence of PD-L1 expression on TC in the context of an activated immune infiltrate as represented by high PD-L1 IC positivity. These findings may help towards a better understanding of the tumor-immune system interaction and also signify the clinical relevance of PD-L1 expression on IC as a biomarker for immunotherapy in NSCLC patients.

## Supporting information

S1 File Supplementary tables. (DOCX)

S1 Fig Examples of PD-L1 co-staining of TC and IC positivity in various subgroups. (ZIP)

S2 Fig Percentages in Venn diagrams represent the overlap of PD-L1 expression of the TC3 with IC3, the TC2 with IC2 and the TC1 with IC1 subgroups. (TIF)

S3 Fig PD-L1 expression on TC and IC and associations with histology, smoking and *KRAS* status. (A, B) No significant difference was seen between SCC compared to AC regarding PD-L1 protein expression on TC or IC (n = 615). (C) PD-L1 protein expression on TC is significantly higher in heavy compared to light smokers (n = 526). (D) No significant difference was seen between heavy compared to light smokers regarding PD-L1 protein expression on IC (n = 526). (E) PD-L1 protein expression on TC is significantly higher in *KRASm* compared to *KRASwt* samples in the AC cohort only (n = 317). (F) No significant difference was seen between *KRASm* compared to *KRASwt* samples regarding PD-L1 expression on IC in the AC cohort only (n = 317). All boxplots were plotted on a hyperlog-transformed y-axis (see Materials and Methods). * p = 0.016, ** p < 0.001, univariate analysis. AC = adenocarcinoma, SCC = squamous cell carcinoma.
(TIFF)

S4 Fig Associations of PD-L1 protein expression on TC and IC in non-overlapping subgroups with mRNA expression of *CD274*. ns = non significant, ** p < 0.01, *** p < 0.001.
(TIFF)

S5 Fig Associations of mRNA expression of *PDCD1*, *PDCD1LG2*, *CD8A* and the T_eff_ signature in non-overlapping PD-L1 expressing subgroups. (A) Relative mRNA expression of *PDCD1*, *PDCD1LG2*, *CD8A* and the T^eff^ signature in TC3 tumors based on various levels of IC (n = 39). (B) Relative mRNA expression of *PDCD1*, *PDCD1LG2*, *CD8A* and the T^eff^ signature in IC3 tumors based on various levels of TC (n = 83). (C) Relative mRNA expression of the *PDCD1*, *PDCD1LG2*, *CD8A* and the T^eff^ signature in TC0 tumors based on various levels of IC (n = 351). ns = non significant, * p = 0.01–0.05, * p < 0.01, *** p < 0.001.
(TIFF)

S6 Fig Expression of the T_eff_ signature vs the expression of the IFNγ response signature. (TIFF)
