# Supplementary material for: Absence of PD-L1 expression on tumor cells in the context of an activated immune infiltrate may indicate impaired IFNγ signaling in non-small cell lung cancer
Source: PLoS One. 2019 May 24;14(5):e0216864. doi: 10.1371/journal.pone.0216864 (PMC6534376; doi:10.1371/journal.pone.0216864)
Supplement: S1 File — (DOCX) [file pone.0216864.s001.docx]

**Supporting information**

**Table A. List of hotspot mutations.**

| Gene | COSMIC ID | cDNA mutation | AA mutation |
| --- | --- | --- | --- |
| EGFR | 6252 | 2155 G>A | G719S |
| EGFR | 6253 | 2155 G>T | G719C |
| EGFR | 6239 | 2156 G>C | G719A |
| EGFR | 26038 | 2233_2247del15 | K745_E749del |
| EGFR | 13550 | 2235_2248>AATTC | E746_A750>IP |
| EGFR | 6223 | 2235_2249del15 | E746_A750del |
| EGFR | 13552 | 2235_2251>AATTC | E746_T751>IP |
| EGFR | 13551 | 2235_2252>AAT | E746_T751>I |
| EGFR | 12385 | 2235_2255>AAT | E746_S752>I |
| EGFR | 12413 | 2236_2248>AGAC | E746_A750>RP |
| EGFR | 6225 | 2236_2250del15 | E746_A750del |
| EGFR | 12728 | 2236_2253del18 | E746_T751del |
| EGFR | 12678 | 2237_2251del15 | E746_T751>A |
| EGFR | 12386 | 2237_2252>T | E746_T751>V |
| EGFR | 12416 | 2237_2253>TTGCT | E746_T751>VA |
| EGFR | 12367 | 2237_2254del18 | E746_S752>A |
| EGFR | 12384 | 2237_2255>T | E746_S752>V |
| EGFR | 18427 | 2237_2257>TCT | E746_P753>VS |
| EGFR | 12422 | 2238_2248>GC | L747_A750>P |
| EGFR | 23571 | 2238_2252del15 | L747_T751del |
| EGFR | 12419 | 2238_2252>GCA | L747_T751>Q |
| EGFR | 6220 | 2238_2255del18 | E746_S752>D |
| EGFR | 6218 | 2239_2247del9 | L747_E749del |
| EGFR | 12382 | 2239_2248TTAAGAGAAG>C | L747_A750>P |
| EGFR | 12383 | 2239_2251>C | L747_T751>P |
| EGFR | 6254 | 2239_2253del15 | L747_T751del |
| EGFR | 6255 | 2239_2256del18 | L747_S752del |
| EGFR | 12403 | 2239_2256>CAA | L747_S752>Q |
| EGFR | 12387 | 2239_2258>CA | L747_P753>Q |
| EGFR | 6210 | 2240_2251del12 | L747_T751>S |
| EGFR | 12369 | 2240_2254del15 | L747_T751del |
| EGFR | 12370 | 2240_2257del18 | L747_P753>S |
| EGFR | 13556 | 2253_2276del24 | S752_I759del |
| EGFR | 6241 | 2303 G>T | S768I |
| EGFR | 12376 | 2307_2308 ins 9(gccagcgtg) | V769_D770insASV |
| EGFR | 13558 | 2309_2310complex(ac>ccagcgtggat) | V769_D770insASV |
| EGFR | 12378 | 2310_2311 ins GGT | D770_N771insG |
| EGFR | 13428 | 2311_2312 ins 9(gcgtggaca) | D770_N771insSVD |
| EGFR | 12377 | 2319_2320 ins CAC | H773_V774insH |
| EGFR | 6240 | 2369 C>T | T790M |
| EGFR | 6224 | 2573 T>G | L858R |
| EGFR | 12429 | 2573-2574TG>GT | L858R |
| EGFR | 6213 | 2582 T>A | L861Q |
| PIK3CA | 746 | c.263G>A | R88Q |
| PIK3CA | 754 | c.1035T>A | N345K |
| PIK3CA | 757 | c.1258T>C | C420R |
| PIK3CA | 760 | c.1624G>A | E542K |
| PIK3CA | 763 | c.1633G>A | E545K |
| PIK3CA | 12458 | c.1634A>C | E545A |
| PIK3CA | 764 | c.1634A>G | E545G |
| PIK3CA | 765 | c.1635G>T | E545D |
| PIK3CA | 766 | c.1636C>A | Q546K |
| PIK3CA | 6147 | c.1636C>G | Q546E |
| PIK3CA | 12459 | c.1637A>G | Q546R |
| PIK3CA | 25041 | c.1637A>T | Q546L |
| PIK3CA | 773 | c.3129G>T | M1043I |
| PIK3CA | 12591 | c.3127A>G | M1043V |
| PIK3CA | 776 | c.3140A>T | H1047L |
| PIK3CA | 775 | c.3140A>G | H1047R |
| PIK3CA | 774 | c.3139C>T | H1047Y |
| PIK3CA | 12597 | c.3145G>C | G1049R |
| KRAS | 522 | c.35G>C | G12A |
| KRAS | 516 | c.34G>T | G12C |
| KRAS | 521 | c.35G>A | G12D |
| KRAS | 517 | c.34G>A | G12S |
| KRAS | 518 | c.34G>C | G12R |
| KRAS | 520 | c.35G>T | G12V |
| KRAS | 532 | c.38G>A | G13D |
| KRAS | 512 | c.34_35GG>TT | G12F |
| KRAS | 533 | c.38G>C | G13A |
| KRAS | 527 | c.37G>T | G13C |
| KRAS | 529 | c.37G>C | G13R |
| KRAS | 528 | c.37G>A | G13S |
| KRAS | 534 | c.38G>T | G13V |
| KRAS | 554 | c.183A>C | Q61H |
| KRAS | 555 | c.183A>T | Q61H |
| KRAS | 549 | c.181C>A | Q61K |
| KRAS | 553 | c.182A>T | Q61L |
| KRAS | 552 | c.182A>G | Q61R |
| BRAF | 473 | c.1798_1799GT>AA | V600K |
| BRAF | 476 | c.1799T>A | V600E |
| NRAS | 565 | c.35G>C | G12A |
| NRAS | 562 | c.34G>T | G12C |
| NRAS | 561 | c.34G>C | G12R |
| NRAS | 563 | c.34G>A | G12S |
| NRAS | 566 | c.35G>T | G12V |
| NRAS | 564 | c.35G>A | G12D |
| NRAS | 575 | c.38G>C | G13A |
| NRAS | 570 | c.37G>T | G13C |
| NRAS | 573 | c.38G>A | G13D |
| NRAS | 569 | c.37G>C | G13R |
| NRAS | 574 | c.38G>T | G13V |
| NRAS | 580 | c.181C>A | Q61K |
| NRAS | 584 | c.182A>G | Q61R |
| NRAS | 583 | c.182A>T | Q61L |
| NRAS | 582 | c.182A>C | Q61P |
| NRAS | 586 | c.183A>C | Q61H |
| NRAS | 585 | c.183A>T | Q61H |
| AKT1 | 33765 | c.49G>A | E17K |
| FLT3 | 785 | c.2503G>C | D835H |
| FLT3 | 783 | c.2503G>T | D835Y |
| FLT3 | 784 | c.2504A>T | D835V |
| FLT3 | 788 | c.2505T>G | D835E |
| HRAS | 480 | c.34G>A | G12S |
| HRAS | 481 | c.34G>T | G12C |
| HRAS | 483 | c.35G>T | G12V |
| HRAS | 484 | c.35G>A | G12D |
| HRAS | 487 | c.37G>A | G13S |
| HRAS | 486 | c.37G>C | G13R |
| HRAS | 496 | c.181C>A | Q61K |
| HRAS | 499 | c.182A>G | Q61R |
| HRAS | 498 | c.182A>T | Q61L |
| HRAS | 503 | c.183G>C | Q61Hc |
| HRAS | 502 | c.183G>T | Q61Ht |
| KIT | 1216 | c.1669T>A | W557R |
| KIT | 1219 | c.1669T>C | W557G |
| KIT | 1290 | c.1727T>C | L576P |
| KIT | 1304 | c.1924A>G | K642E |
| KIT | 12706 | c.1961T>C | V654A |
| KIT | 1311 | c.2446G>C | D816H |
| KIT | 1310 | c.2446G>T | D816Y |
| KIT | 1314 | c.2447A>T | D816V |
| MET | 710 | c.1124A>G | N375S |
| MET | 707 | c.3029C>T | T1010I |
| MET | 699 | c.3743A>G | Y1248C |
| MET | 700 | c.3757T>G | Y1253D |
| JAK2 | 12600 | c.1849G>T | V617F |
| MYD88 | 85940 | c.794T>C | L256P |
| ERBB2 | 14060 | c.2264T>C | L755S |
| ERBB2 | 683 | c.2263_2264TT>CC | L755P |
| ERBB2 | 14062 | c.2329G>T | L777L |

**Table B. Clinicopathological features in TC3/IC<3 vs TC<3/IC3 samples.**

|  | **TC3/IC<3** | **TC<3/IC3** | **p-value** |
| --- | --- | --- | --- |
| **Total (n = 125)** | 36 | 89 |  |
| **Gender** |  |  |  |
| Male | 15 (41.7%) | 45 (50.6%) |  |
| Female | 21 (58.3%) | 44 (49.5%) | .43 |
|  |  |  |  |
| **Median age at surgery (years, range)** | 59 (39-77) | 64 (36-82) | .057 |
|  |  |  |  |
| **Smoking** |  |  |  |
| Light smokers <10PY | 1 (2.8%) | 0 |  |
| Heavy smokers ≥10PY | 32 (88.9%) | 70 (78.7%) |  |
| Unknown | 3 (8.3%) | 19 (21.3%) | .32 |
|  |  |  |  |
| **Histology** |  |  |  |
| Adenocarcinoma | 15 (41.6%) | 52 (58.4%) |  |
| Squamous cell carcinoma | 20 (55.6%) | 33 (37.1%) |  |
| NSCLC NOS | 1 (2.8%) | 4 (4.5%) | .14 |
|  |  |  |  |
| **Tumor stage at resection** |  |  |  |
| Stage I | 21 (58.3%) | 40 (45.0%) |  |
| Stage II | 9 (25.0%) | 30 (33.7%) |  |
| Stage III | 5 (13.9%) | 17 (19.1%) |  |
| Stage IV | 1 (2.8%) | 2 (2.3%) | .29 |
|  |  |  |  |
| **Genetic alterations** |  |  |  |
| *EGFRm* | 0 | 2 (2.2%) | .09 |
| *KRASm* | 11 (30.1%) | 19 (21.3%) | .71 |
|  |  |  |  |
