# Supplementary figures and images for: Absence of PD-L1 expression on tumor cells in the context of an activated immune infiltrate may indicate impaired IFNγ signaling in non-small cell lung cancer
Source: PLoS One. 2019 May 24;14(5):e0216864. doi: 10.1371/journal.pone.0216864 (PMC6534376; doi:10.1371/journal.pone.0216864)

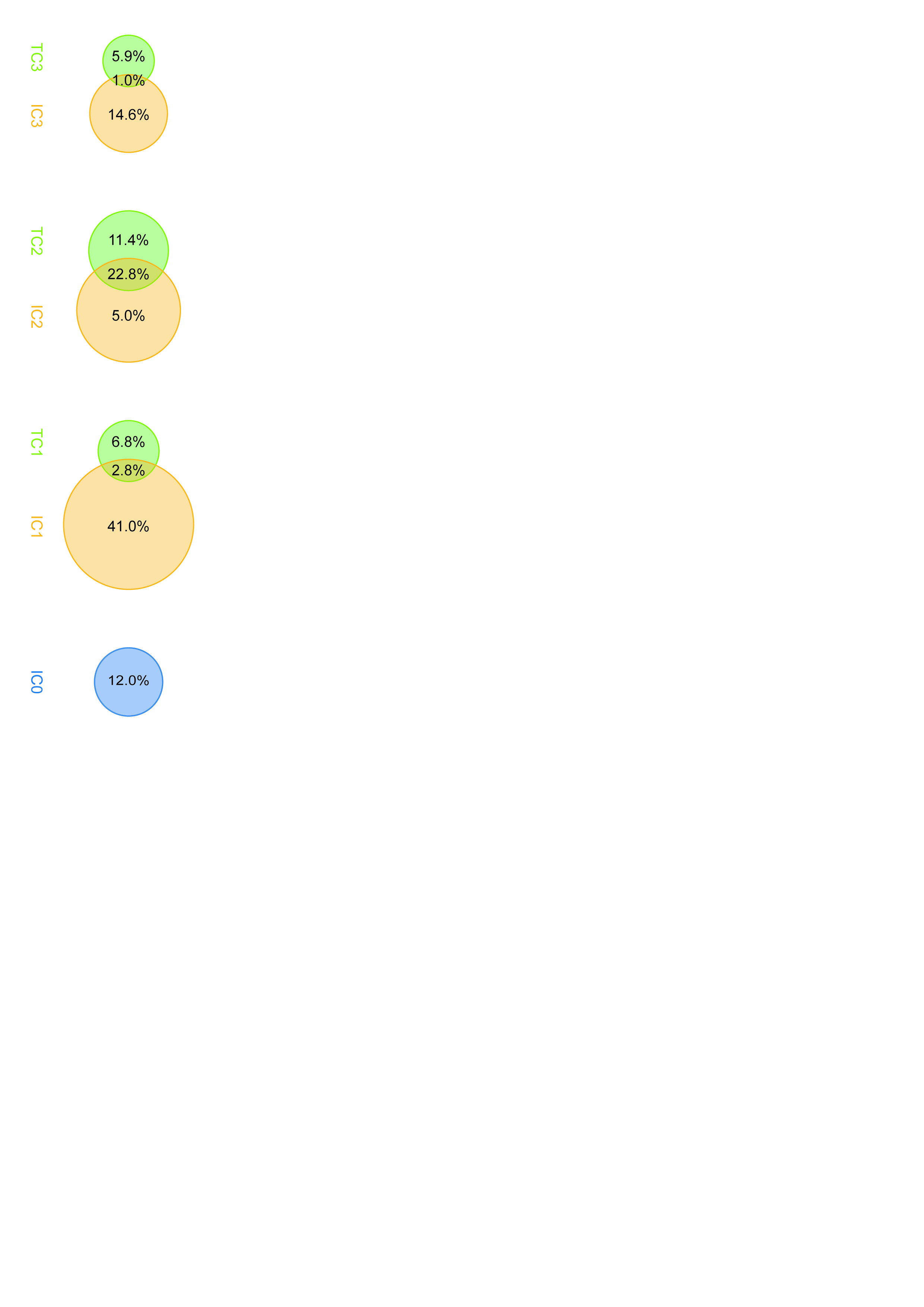

Supplement: S2 Fig — (TIF) [file pone.0216864.s003.tif]

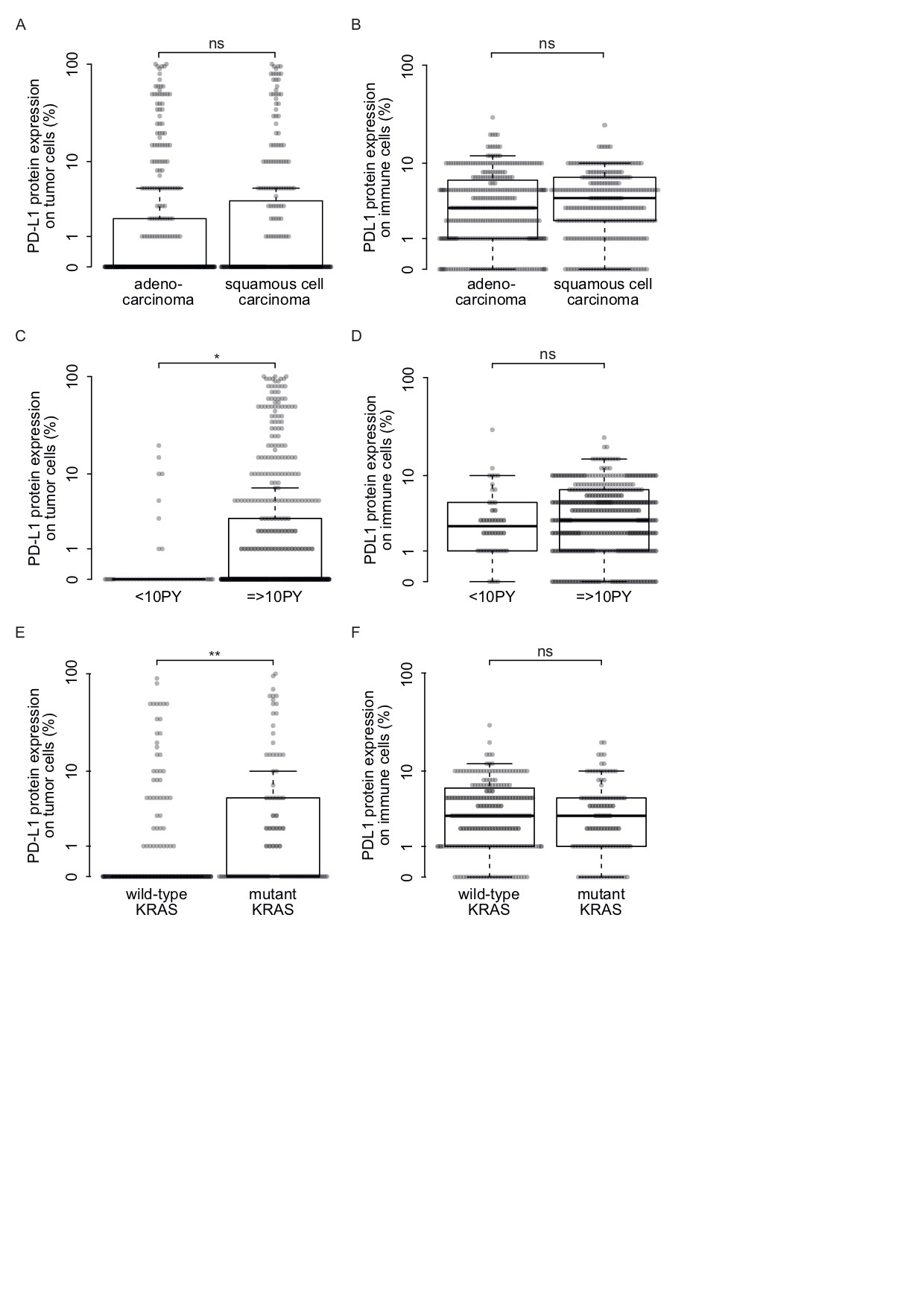

Supplement: S3 Fig — (A, B) No significant difference was seen between SCC compared to AC regarding PD-L1 protein expression on TC or IC (n = 615). (C) PD-L1 protein expression on TC is significantly higher in heavy compared to light smokers (n = 526). (D) No significant difference was seen between heavy compared to light smokers regarding PD-L1 protein expression on IC (n = 526). (E) PD-L1 protein expression on TC is significantly higher in KRASm compared to KRASwt samples in the AC cohort only (n = 317). (F) No significant difference was seen between KRASm compared to KRASwt samples regarding PD-L1 expression on IC in the AC cohort only (n = 317). All boxplots were plotted on a hyperlog-transformed y-axis (see Materials and Methods). * p = 0.016, ** p < 0.001, univariate analysis. AC = adenocarcinoma, SCC = squamous cell carcinoma. (TIFF) [file pone.0216864.s004.tiff]

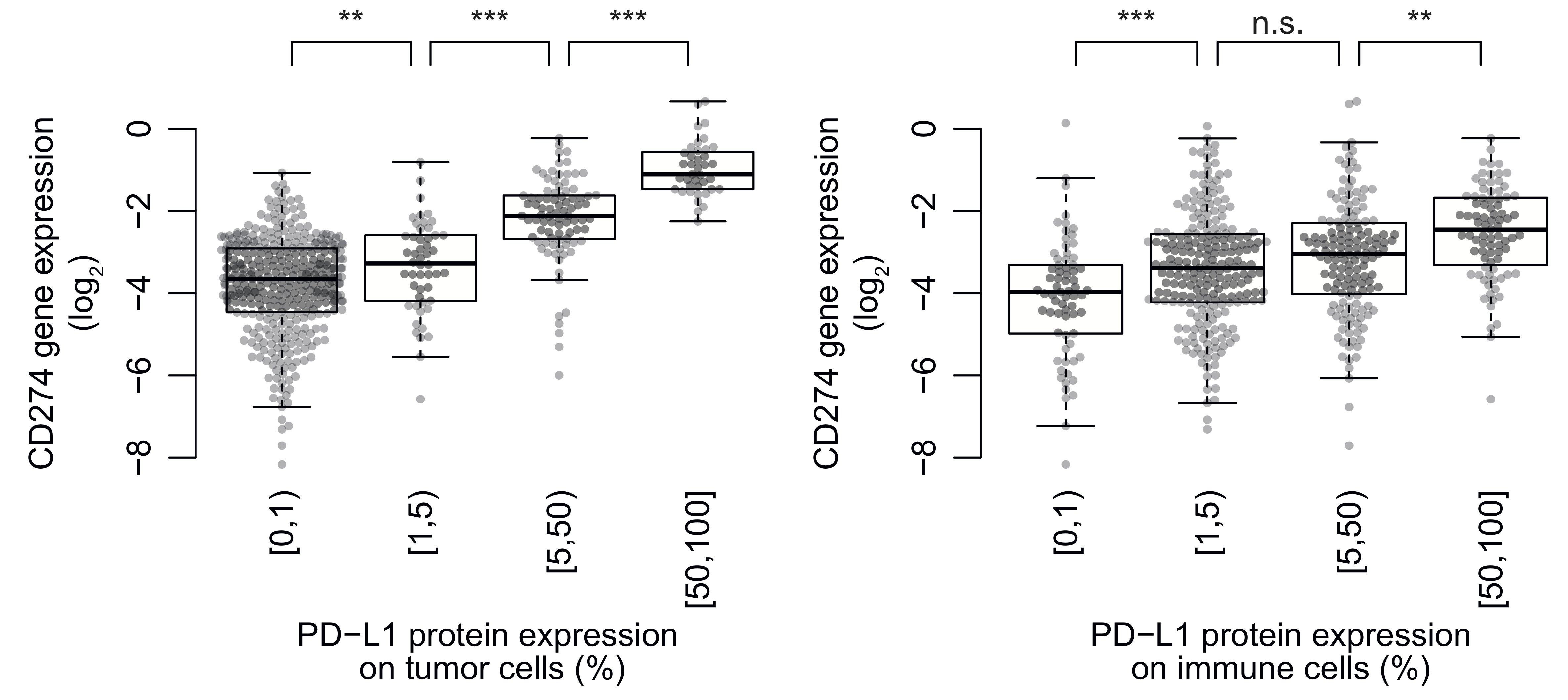

Supplement: S4 Fig — ns = non significant, ** p < 0.01, *** p < 0.001. (TIFF) [file pone.0216864.s005.tiff]

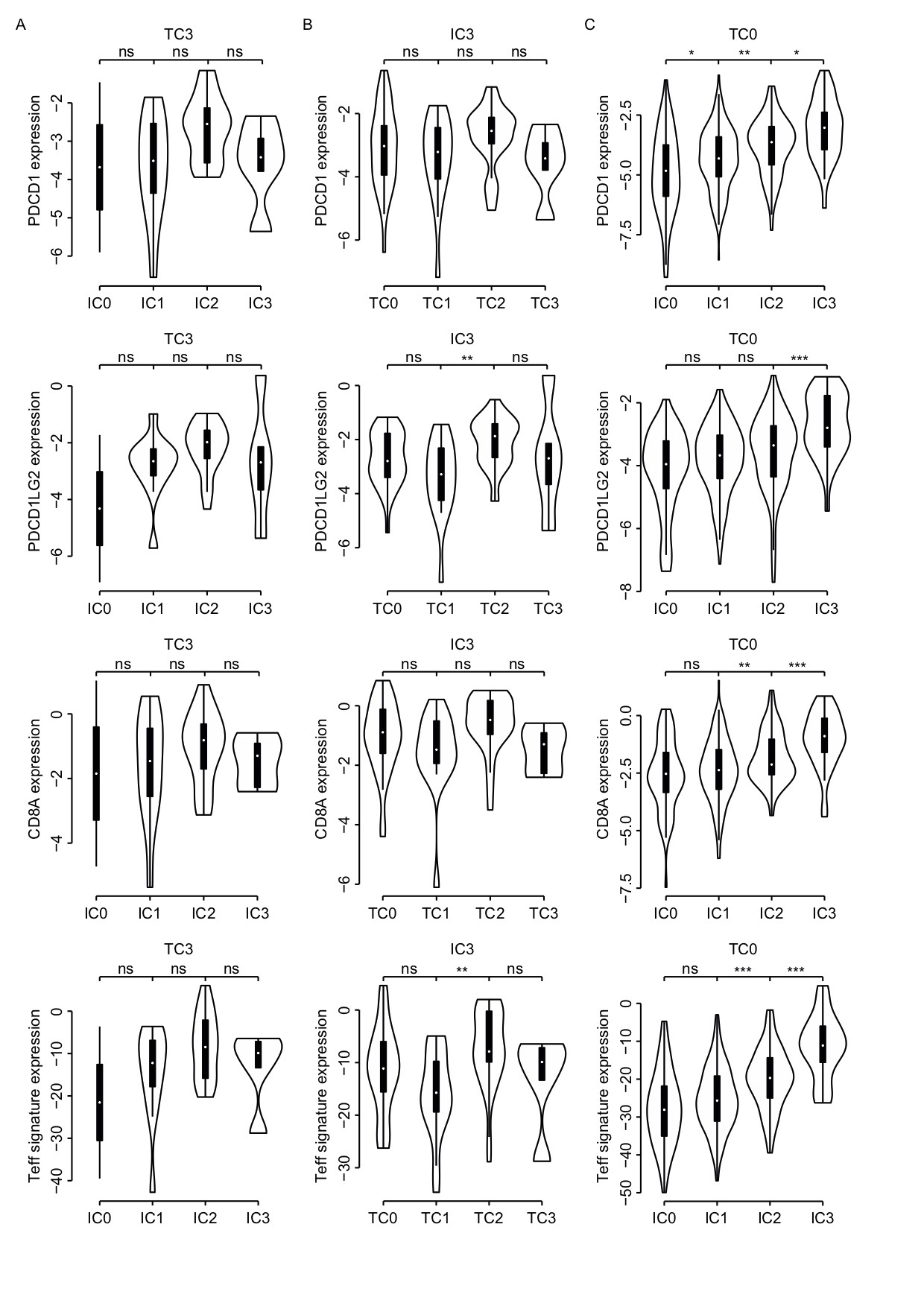

Supplement: S5 Fig — (A) Relative mRNA expression of PDCD1, PDCD1LG2, CD8A and the Teff signature in TC3 tumors based on various levels of IC (n = 39). (B) Relative mRNA expression of PDCD1, PDCD1LG2, CD8A and the Teff signature in IC3 tumors based on various levels of TC (n = 83). (C) Relative mRNA expression of the PDCD1, PDCD1LG2, CD8A and the Teff signature in TC0 tumors based on various levels of IC (n = 351). ns = non significant, * p = 0.01–0.05, * p < 0.01, *** p < 0.001. (TIFF) [file pone.0216864.s006.tiff]

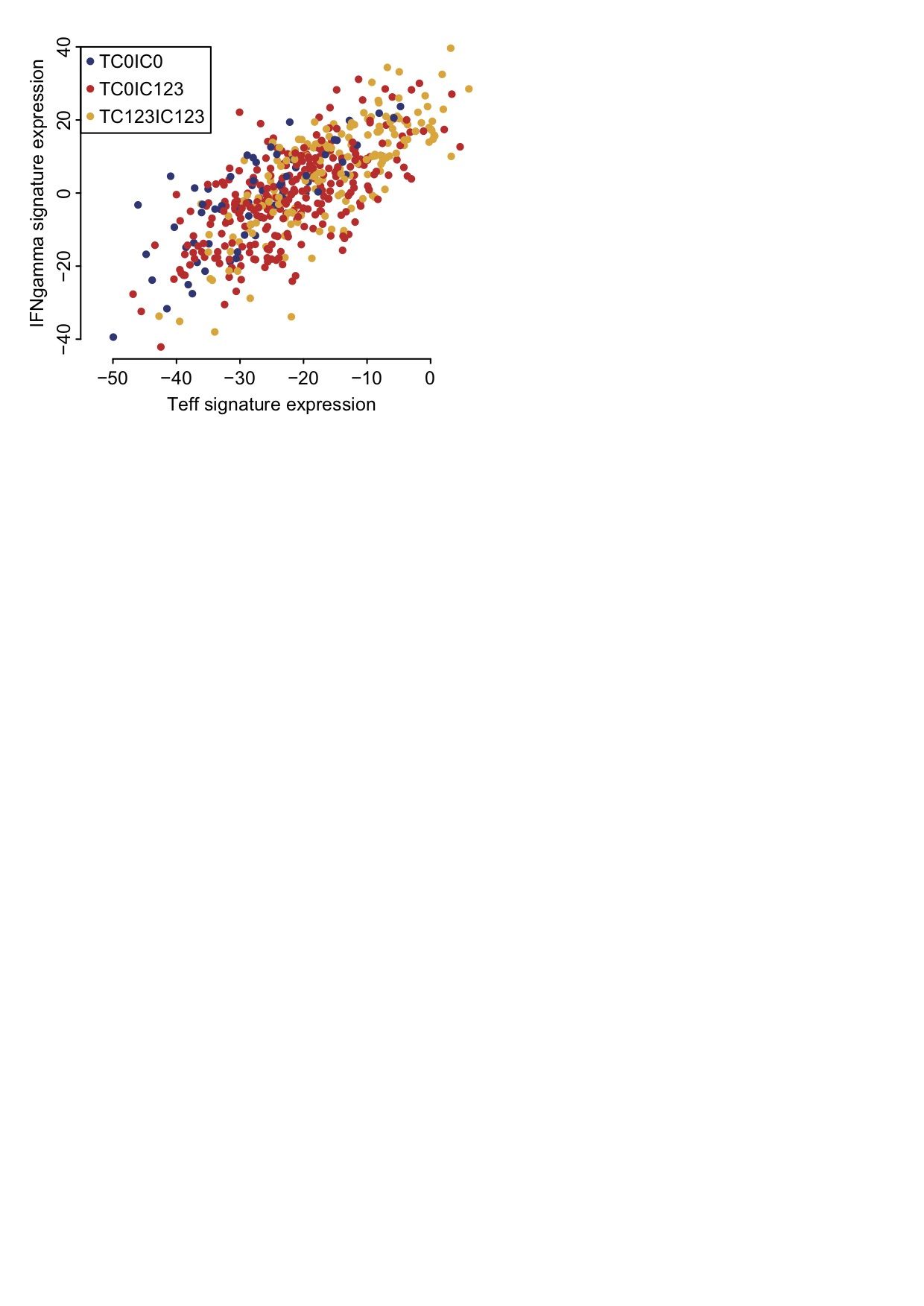

Supplement: S6 Fig — (TIFF) [file pone.0216864.s007.tiff]
